# Wearable sensor measurements in relation to clinical characteristics and mortality in patients with Parkinson’s disease

**DOI:** 10.1186/s42466-026-00474-8

**Published:** 2026-03-11

**Authors:** Daniel von Below, Susanna M. Wallerstedt, Filip Bergquist

**Affiliations:** 1 Department of Clinical Neuroscience, Sahlgrenska Academy, University of Gothenburg, Blå stråket 7, Gothenburg, 413 45 Sweden; 2 Department of Neurology, Sahlgrenska University Hospital, Region Västra Götaland, Gothenburg, Sweden; 3 Department of Pharmacology, Sahlgrenska Academy, University of Gothenburg, Gothenburg, Sweden; 4 Centre for Health Technology Assessment, Sahlgrenska University Hospital, Region Västra Götaland, Gothenburg, Sweden

**Keywords:** Parkinson’s disease, Outpatient monitoring, Bradykinesia, Mortality, Prognostic factors

## Abstract

**Background:**

Parkinson’s disease (PD) is highly variable between patients, and regular assessments are needed to adjust symptomatic treatment. Wearable sensor measurements can complement clinical examinations and patient-reported outcome measures in the management of PD, but their clinical usefulness is yet to be established. Previous studies have described wearable sensor measurements from selected patients, often with advanced disease, but not PD patients in general. We sought to objectively describe daily-life movement characteristics of population-representative patients with PD using the wrist-worn Personal KinetiGraph (PKG), and to relate these sensor measurements to clinical data and proposed PKG-based treatment targets.

**Methods:**

Individuals in a population-based random sample of patients with PD were evaluated with clinical assessments, patient-reported outcome measures and six-day PKG recordings. PKG outcomes included bradykinesia score (BKS) and dyskinesia score (DKS), reflecting symptom severity. Mortality within eight years was recorded.

**Results:**

The study included 286 patients with a median age of 73 years and a median time since diagnosis of five years. Clinical and patient-reported variables expected to increase with disease or symptom severity were positively correlated with bradykinesia score (BKS) and negatively correlated with dyskinesia score (DKS). Patients with BKS > 25 had lower levodopa-equivalent daily dose, higher self-reported symptom burden and lower health-related quality of life compared to patients with BKS ≤ 25. Age- and sex-adjusted mortality rates were 1.8 times higher in patients with BKS > 25.

**Conclusions:**

Sensor-based motor assessments with PKG reflect clinical outcome measures in population-representative PD patients. A high degree of sensor-assessed bradykinesia (BKS > 25) at baseline was associated with higher mortality, shorter disease duration and less intensive treatment. The results suggest that high BKS is a risk factor for mortality, possibly as a marker of undertreatment.

**Trial registration:**

ClinicalTrials.gov identifier NCT03130595, registration date 22 April 2017.

**Supplementary Information:**

The online version contains supplementary material available at 10.1186/s42466-026-00474-8.

## Background

Parkinson’s disease (PD) is highly variable between patients, both in terms of symptom prevalence and rate of progression [1], and regular assessments are therefore needed to adjust symptomatic treatment to the individual patient. Motor symptoms—such as bradykinesia, tremor, and rigidity—generally respond to dopamine replacement therapy, but eventually most patients will require a complex regimen involving multiple drug classes [2, 3]. The traditional approach to assess motor symptoms is to combine patient history with clinical examination, sometimes using systematic rating scales like the Movement Disorder Society-Sponsored Revision of the Unified Parkinson’s Disease Rating Scale (MDS-UPDRS) [4]. However, patient history is susceptible to bias, and clinical examination only provides a snapshot of the patient’s motor function. This approach may therefore fail to capture treatment response and functioning in daily-life activities. To address these limitations, long-term unsupervised monitoring has been proposed as a complement to clinical assessments [5]. Still, despite consensus on frameworks for digital mobility outcomes, the role of wearable sensors in routine PD care remains uncertain [6, 7].

The first sensor for PD monitoring that gained Food and Drug Administration clearance was the Personal KinetiGraph (PKG; also called Parkinson’s KinetiGraph) and several other sensors are now available [6]. The PKG measures include bradykinesia score (BKS), dyskinesia score (DKS), fluctuation dyskinesia score (FDS), percent time immobilized (PTI) and percent time with tremor (PTT), all of which have been validated separately [8–11]. De-identified PKG measurement data have been published from the global use of PKG in clinical routine [12] and there are several observational studies that describe experiences from movement disorder clinics using PKG in selected patient populations [13–15]. However, little is known about sensor measurements in unselected patients with PD. Our experience from clinical routine is that patients are usually referred for PKG when they reach a stage of disease where device-assisted therapies are considered, which means that the previously published measurement data may not be representative of patients with PD in general. Another issue regarding the use of wearable sensors for PD monitoring has been the lack of established treatment targets. In the case of PKG, preliminary treatment targets have been proposed by a movement disorder specialist panel, where BKS > 25 is interpreted as “uncontrolled” bradykinesia and DKS > 9 as “uncontrolled” dyskinesia [16]. A single-blinded trial (*n* = 154) has reported better effects on motor outcome when therapeutic decisions were guided by PKG measurement targets, compared to when decisions were based on clinical assessment alone [17]. However, while treat-to-target strategies are appealing theoretically, the clinical usefulness of the abovementioned treatment targets for BKS and DKS has not been clearly demonstrated, and there is limited information on how these treatment targets relate to clinical outcomes.

To address some of these questions, we have evaluated results of PKG measurements in a random patient sample from Region Västra Götaland, Sweden. The aims of this study were to objectively describe motor characteristics of population-representative PD patients in their daily lives, and to explore the association between wearable sensor measurements and other clinical data—including mortality—in relation to proposed PKG targets for controlled bradykinesia and dyskinesia.

## Methods

Data were obtained from the longitudinal observational study West Sweden Parkinson Objective Measurement Registry Study (ClinicalTrials.gov identifier NCT03130595). Ethics approval was obtained from the Regional Ethical Review Board in Gothenburg, Sweden (122 − 17). All participants provided written informed consent.

### Study population

Patients were recruited among all individuals with a recorded PD diagnosis (G20.9; International Classification of Diseases, 10th Revision) and a visit to any of the eight outpatient clinics with PD-specialized physicians (neurologists or geriatricians) within public healthcare in Region Västra Götaland (population: 1.7 million) during two consecutive six-month periods between September 2016 and April 2018. For each clinic and period, every fourth patient was selected from a list of patients arranged by birth date, with the starting number (1–4) determined by a pseudorandom number generator. Using this procedure, a random sample representing 25% of the patients were invited to participate. Patients who accepted participation were included unless the diagnosis had been changed before the study assessment visit. The population has previously been described in a validation study of a patient-reported outcome measure for PD [18].

### Wearable sensor measurements

Worn like a wristwatch, PKG measures velocity changes of the wrist for a set amount of time, usually six consecutive days. Recordings are divided into two-minute epochs that include mean spectral power, peak acceleration, and time without movement. Built-in algorithms use these parameters to calculate BKS and DKS, with the general assumption that bradykinesia corresponds to movements with lower acceleration and amplitude, as well as longer intervals between movements, and that dyskinesia corresponds to movements with normal amplitude and acceleration, but with shorter intervals between movements. In early validation studies, BKS had a positive correlation with Part III of the Unified Parkinson’s Disease Rating Scale and DKS had a positive correlation with the Abnormal Involuntary Movement Scale; both were responsive to changes in medication [11]. Later FDS, PTI, and PTT were added as separate outcome measures, which have been validated separately [8–10]. FDS is calculated from the interquartile ranges of BKS and DKS [8]. PTI represents the percentage of two-minute epochs with BKS ≥ 80 [9]. PTT represents the percentage of two-minute epochs where a specific algorithm has detected tremor [10].

### Study procedures

Between 2017 and 2018, study participants underwent an on-site study visit and received a PKG device which was returned by mail after it had been worn for six full consecutive days and nights. BKS, DKS, FDS, PTI, and PTT were registered for all patients. As described earlier, we used previously defined cut-off values to define “uncontrolled” levels of bradykinesia (BKS > 25) and “uncontrolled” levels of dyskinesia (DKS > 9) [16]. We refrained from dichotomizing FDS, PTI and PTT since there are no established cut-off values.

The study visits included clinical assessments and the collection of demographic and clinical data as well as patient-reported outcome measures. The Clinical Impression of Severity Index for Parkinson’s Disease (CISI-PD) [19, 20] was administered by a trained study nurse with extensive experience of patients with PD. Blood pressure was measured in sitting and standing. Orthostatic hypotension was defined as (1) a drop in systolic blood pressure ≥ 20 mmHg, (2) a drop in diastolic blood pressure ≥ 10 mmHg, or (3) a systolic blood pressure in standing < 90 mmHg [21]. The levodopa-equivalent daily dose (LEDD) was calculated for each patient according to established formulae; anticholinergic drugs (used by six patients) were not included as it could not be determined whether each dose provided clinically important improvement, a requirement for inclusion of these drugs [22]. Four patient-reported outcome measures were collected, either on paper or online on the Swedish Parkinson Registry website: Patient-Reported Outcomes in Parkinson’s Disease (PRO-PD) [18, 23], the Non-Motor Symptoms Questionnaire (NMSQ) [24], the EuroQoL Five-Dimension Five-Level Scale (EQ-5D-5 L) [25, 26], and the Eight-Item Parkinson’s Disease Questionnaire Summary Index (PDQ8-SI) [27, 28].

### Mortality

All study participants who died up to October 2025 were identified using data from the Swedish Parkinson Registry, which is updated twice a year with data from the Swedish Population Register. The observation period for living patients ended on the date of the last recorded death, to remove the effects of potential reporting delays. Standardized mortality ratio (SMR) was calculated using official numbers for Region Västra Götaland from Statistics Sweden.

### Clinical referral population

To investigate potential differences between PKG measurements in the studied random sample of PD patients and PKG measurements in clinical practice, we compiled data from all routine PKG recordings performed for clinical reasons at Sahlgrenska University Hospital during the period when the study recordings took place, i.e., between January 2017 and December 2018. These de-identified data have previously contributed to a large, international descriptive study [12].

### Statistics

Calculations were performed using IBM SPSS Statistics (version 28). Spearman’s rank test was used to obtain correlation coefficients with a two-tailed test of significance. Comparisons between groups were made with the Mann-Whitney U test (continuous variables) or Fisher’s exact test (categorical variables). Significance levels were set to *p* < 0.05. Unless otherwise stated, data are presented as median with first and third quartiles within brackets.

SMR was calculated as the ratio of observed to expected deaths. The number of expected deaths was calculated by applying the mortality rates of the general population in Region Västra Götaland, stratified by sex and five-year age-bands, to corresponding groups of the study population at risk for each year between 2017 and 2025 [29]. Patients were included in the population at risk each year that they were alive at mid-year, starting from their first study visit. Mortality rates for 2024 were used also for 2025 since official numbers were not available. Byar’s approximation of Poisson distribution was used to calculate the 95% confidence intervals (CI) of the SMR [30]. Mortality rates were calculated by dividing the number of deaths by the number of person-years at risk. Age- and sex-adjusted mortality rates were calculated by direct adjustment, using 20-year age-bands and the whole study population as the standard [29]. Survival by BKS status (> 25 or ≤ 25) was described using a Kaplan-Meier plot, where the time variable was the number of days between the assessment visit and death, or between the assessment visit and the censoring date.

## Results

**Fig. 1:**
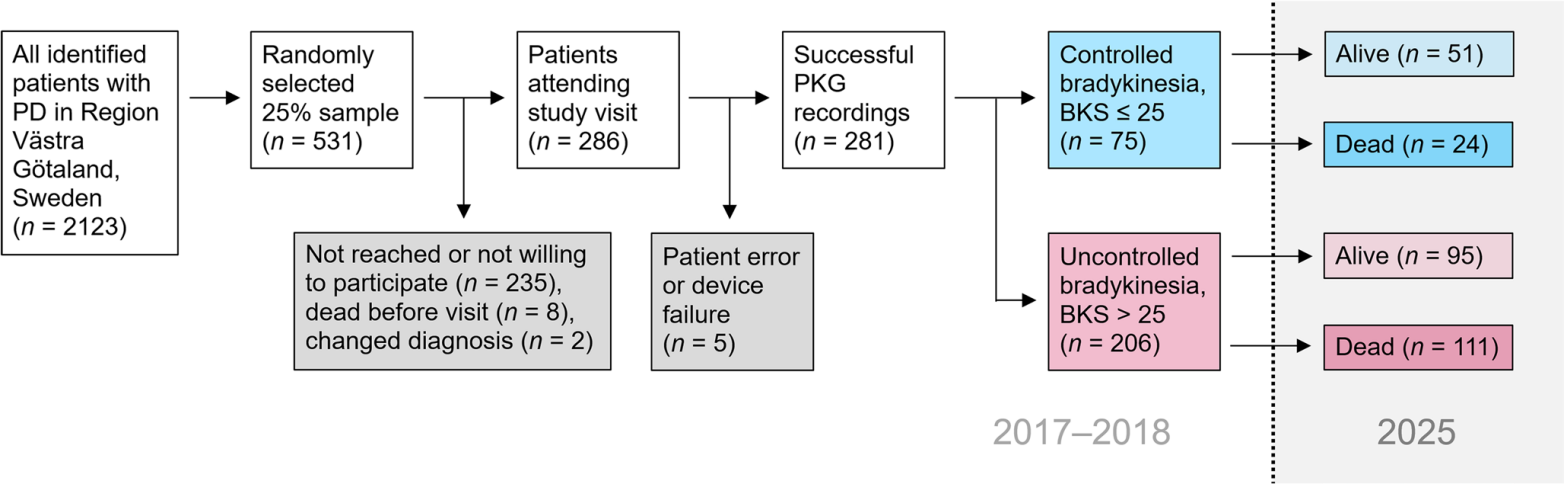
Flowchart of study participants. Baseline data were collected in 2017–2018, including PKG measurements, and mortality was analyzed in 2025

Between 2017 and 2018, we identified 2,123 eligible patients with PD and invited a random sample of 531, out of whom 286 were included in the study (Fig. 1). The study population had a median age of 73 years (66–78) and 65% were men. Clinical characteristics are presented in Table 1. In comparison, the 235 invited but nonparticipating patients were older (74 (68–80) years, *p* = 0.003) and the proportion of men was lower (52%, *p* = 0.006).

Mortality was recorded for the whole study population up to October 2025. During this eight-year period, 135 of 286 patients died. The unadjusted mortality rate was 74 deaths per 1000 person-years. Using mortality rates of the general population for the same region and time period, the expected number of deaths in the study population was 56, resulting in an SMR of 2.4 (95% CI: 2.0 to 2.8).

**Table 1:** Patient characteristics

	**All participants** **(*n*** ** = 286)**	**Participants with BKS ≤ 25** **(*n*** ** = 75)**	**Participants with BKS > 25** **(*n*** ** = 206)**	***p*** ** value**
Age, y median (Q1–Q3)	72.8 (65.9–77.6)	71.2 (62.8–76.1)	73.6 (67.5–78.0)	0.038
Sex, *n* male / female (% male)	187 / 99 (65.4)	36 / 39 (48.0)	148 / 58 (71.8)	< 0.001
Living alone, *n* (%)	61 (21.3)	20 (26.7)	40 (19.4)	0.192
Treatment at non-university hospital, *n* (%)	193 (67.5)	52 (69.3)	137 (66.5)	0.774
Time since diagnosis, y median (Q1–Q3)	5.3 (2.6–10.5)	7.2 (2.8–13.6)	4.9 (2.3–8.6)	0.010
Orthostatic hypotension, *n* (%)*	36 (15.1)	8 (12.3)	28 (16.5)	0.545
LEDD, mg median (Q1–Q3)	600 (400–953)	831 (500–1250)	576 (400–900)	0.002
Conventional treatment, *n* (%)				
Levodopa	272 (95.1)	73 (97.3)	194 (94.2)	0.366
Dopamine agonist	156 (54.5)	50 (66.7)	103 (50.0)	0.015
COMT inhibitor	57 (19.9)	22 (29.3)	35 (17.0)	0.029
MAO-B inhibitor	56 (19.6)	20 (26.7)	36 (17.5)	0.094
Amantadine	13 (4.5)	6 (8.0)	7 (3.4)	0.116
Anticholinergic drug	6 (2.1)	1 (1.3)	5 (2.4)	1.000
Device-assisted treatment, *n* (%)**	15 (5.2)	9 (12.0)	6 (2.9)	0.005
CISI-PD, median (Q1–Q3)*	9 (6–11)	8 (6–10)	9 (6–12)	0.280
Self-reported symptom burden, median (Q1–Q3)				
PRO-PD*	995 (613–1399)	875 (606–1243)	1036 (641–1460)	0.034
NMSQ*	10 (6–14)	10 (6–13)	10 (6–14)	0.593
Self-reported HRQOL, median (Q1–Q3)				
EQ-5D-5L*	0.71 (0.54–0.84)	0.78 (0.65–0.87)	0.69 (0.49–0.84)	0.007
PDQ8-SI*	25 (13–39)	19 (13–31)	25 (13–41)	0.020

Comparisons between participants with BKS ≤ 25 and participants with BKS > 25 (rightmost column) are performed with Mann-Whitney U test and Fisher’s exact test, as appropriate. For CISI-PD, PRO-PD, NMSQ and PDQ8-SI, higher values denote more severe disease. For EQ-5D-5L, lower values denote more severe disease. *Data available for: Blood pressure measurements (*n* = 238), CISI-PD (*n* = 283), PRO-PD (*n* = 266), NMSQ (*n* = 273), EQ-5D-5L (*n* = 283), PDQ8-SI (*n* = 285). **Deep brain stimulation, levodopa-carbidopa intestinal gel, or subcutaneous apomorphine infusion

### PKG recordings and comparisons with other assessments

Successful six-day PKG recordings were obtained from 281 patients (98%) (Table 2). Missing recordings were explained by patient error or device failure. Most patients (*n* = 260) commenced PKG recordings directly after the assessment visit, and the remainder (*n* = 21) had a median time between the assessment visit and the start of recording of 7 days (3–13).

Comparisons between PKG measures and demographic variables, clinical variables and self-reported variables are presented in Supplementary Table 1 (continuous variables) and Supplementary Table 2 (dichotomous variables). Age and male sex were positively correlated with BKS and PTI, and negatively correlated with DKS and FDS. Neither BKS nor DKS correlated with time since diagnosis. Generally, variables indicating a more severe disease burden, such as clinician-rated disability and self-reported difficulties with activities of daily living, were positively correlated with BKS and negatively correlated with DKS. All correlations were weak (Spearman’s rank correlation coefficient (r_S_) < 0.4) [31] except for the correlation between PTT and self-reported tremor (r_S_ = 0.52).

### Patients with controlled versus uncontrolled PKG measures

The proposed limit for controlled bradykinesia (BKS ≤ 25) [16] was exceeded in 206 patients (73%). Compared to patients with BKS ≤ 25, patients with BKS > 25 were somewhat older and had a shorter time since diagnosis. They also had a lower LEDD, a higher self-reported symptom burden, as measured with PRO-PD, and a lower health-related quality of life, as measured with PDQ8-SI and EQ-5D-5 L (Table 1).

**Fig. 2:**
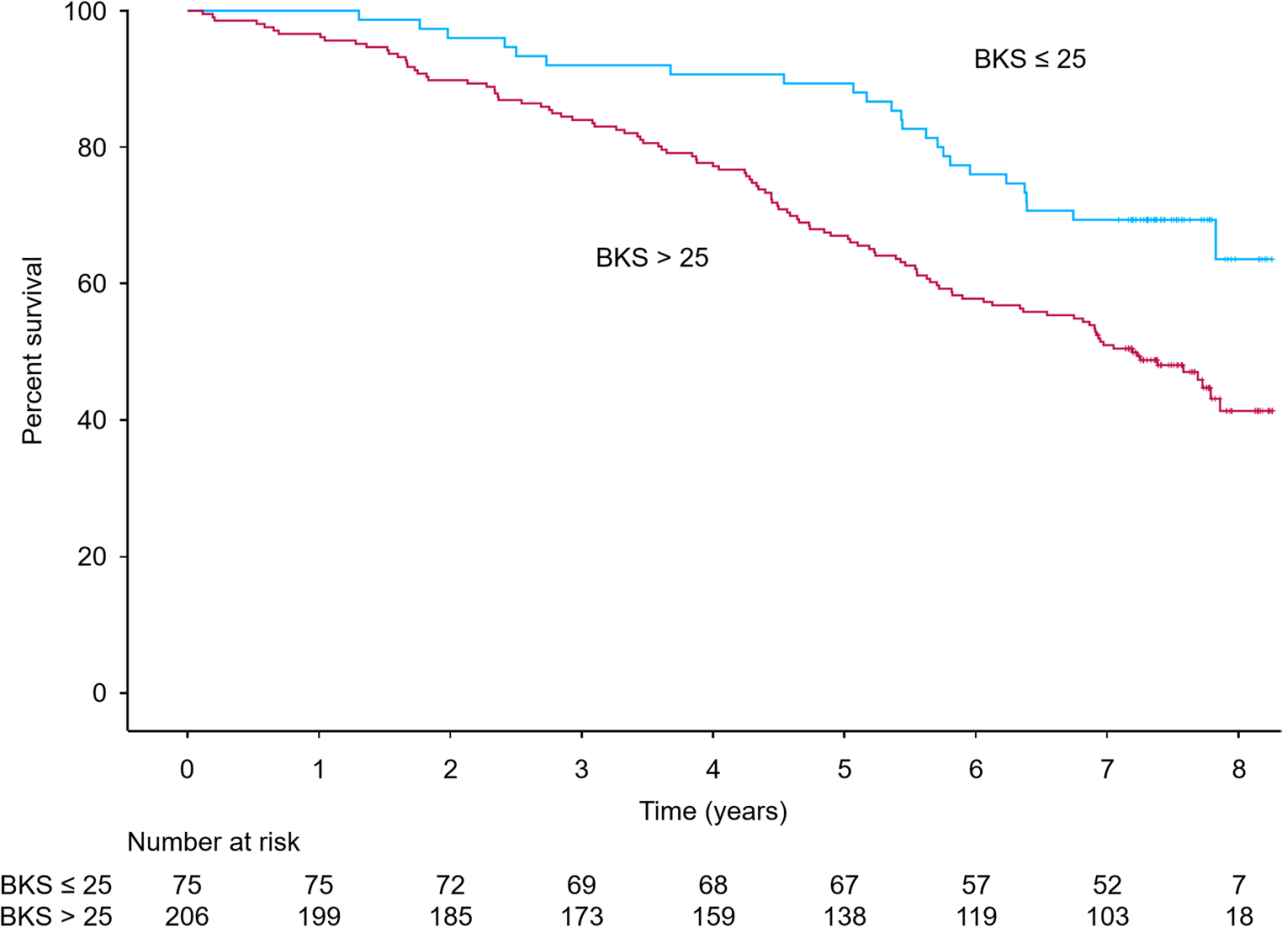
Kaplan-Meier plot of survival for participants with BKS ≤ 25 and participants with BKS > 25

By October 2025, 111 of 206 patients (54%) with BKS > 25 had died, compared to 24 of 75 patients (32%) with BKS ≤ 25 (*p* = 0.001). Kaplan-Meier curves for each group, including numbers at risk, are presented in Fig. 2. The age- and sex-adjusted mortality rate in patients with BKS > 25 was 85 deaths per 1000 person-years (unadjusted 89 deaths per 1000 person-years) and the age- and sex-adjusted mortality rate in patients with BKS ≤ 25 was 47 deaths per 1000 person-years (unadjusted 42 deaths per 1000 person-years), resulting in an age- and sex-adjusted mortality rate ratio of 1.8 (unadjusted 2.1) for high BKS compared to low.

The proposed limit for controlled dyskinesia (DKS ≤ 9) [16] was exceeded in 20 patients (7%), five of whom had died by October 2025. Due to the low number of patients, it was not meaningful to compare mortality or other variables between patients with DKS ≤ 9 and patients with DKS > 9.

### Comparisons with the clinical referral population

During the period of study baseline data collection (2017‒2018), 343 patients had performed PKG recordings on clinical grounds at Sahlgrenska University Hospital. Their anonymized PKG data were available for comparisons. Patients in the clinical referral population had lower BKS, lower PTT, higher DKS, and higher FDS than the study population (Table 2).

**Table 2:** PKG measurements

		**Study population** **(*n*** ** = 281)**	Clinical referral population* **(*n*** ** = 343)**	***p*** ** value**
BKS	median (range)	29.8 (14.2–66.9)	26.6 (8.9–56.4)	< 0.001
	proportion > 25 (%)	73	58	
DKS	median (range)	1.2 (0–35.6)	1.8 (0–58.3)	< 0.001
	proportion > 9 (%)	7	10	
FDS	median (range)	7.7 (2.9–22.3)	8.8 (3.1–32.7)	< 0.001
PTI	median % (range %)	8.3 (0.1–67.9)	7.6 (0–96.4)	0.155
PTT	median % (range %)	2.0 (0.1–49.7)	1.6 (0–47.9)	0.002

*Anonymized data from all patients who performed PKG on clinical grounds in the study region 2017‒2018

## Discussion

This observational study provides objective accelerometry-based measurements of daily-life movement characteristics in a representative sample of patients with PD. Clinically important observations include notably hypokinetic movement patterns, compared to patients performing PKG on clinical indication. Furthermore, patients with sensor-defined uncontrolled bradykinesia had a higher eight-year mortality, despite shorter disease duration and lower treatment intensity.

The PKG measurements from this study provide new insights into the motor characteristics of patients with PD during their daily lives. Both BKS and PTI were notably high in our study population, and only 27% met the criteria for controlled bradykinesia according to suggested objective treatment targets [16]. Recruitment of randomly selected PD patients followed by neurologists and a fair participation rate of 55% resulted in a study sample demographically similar to a recent nationwide Norwegian prevalence study [32]. Measurements can therefore be regarded as representative of patients with PD in Region Västra Götaland, Sweden. Results indicate more hypokinesia in these patients than observed in a previous population-based study from North Tasmania (*n* = 103), where the median BKS was 26.2 and median DKS 1.6 [33]. Contributing explanations include shorter disease duration and possible recruitment bias (40% participation) in the Tasmanian cohort, which was otherwise similar to ours demographically and regarding treatment intensity.

We also report on PKG measurements from patients who were referred for PKG on clinical grounds during the same period. Interestingly, the clinical referral population, which was from the same uptake area, had less hypokinetic movement patterns with a median BKS of 26.6 and a median DKS of 1.8, resembling those of an international sample of nearly 28,000 clinically referred patients [12]. Clinical referral is often prompted by motor fluctuations, which typically emerge in patients with a clear levodopa response. Conversely, our broader sample may include undertreated or less treatment-responsive patients. Comparisons with British [14] and Chinese [15] cohorts further highlight substantial variability in BKS and DKS across regions. The British study reported high BKS (29.6) in early patients with a median LEDD of 375 mg [14], and the Chinese study of PD patients with a median disease duration of five years found median BKS as high as 34.0 [15]. As indicated by a recent international multicenter study [34], these disparities likely reflect geographic and medico-cultural differences in treatment intensity rather than disease progression alone.

This view is further supported by our comparisons between PKG measures and clinical variables. There was a negative correlation between LEDD and BKS, while the correlation between LEDD and DKS was positive. Correspondingly, clinician-rated disability and self-reported difficulties with activities of daily living, were positively correlated with BKS and negatively correlated with DKS. In contrast, neither BKS nor DKS correlated with time since diagnosis. Therefore elevated BKS appears to be a marker for suboptimal treatment. Patients with BKS > 25 had lower LEDD but higher self-reported disease burden, suggesting this threshold effectively identifies undertreated individuals. Assessments with Clinical Impression of Severity Index for Parkinson’s Disease (CISI-PD) did not show significant differences between patients with BKS ≤ 25 and patients with BKS > 25, possibly because a week-long sensor measurement better reflects subjectively experienced disease burden than a snap-shot assessment during follow-up visits. PTI and FDS relate to clinical outcomes in a way that resembles BKS and DKS, respectively. PTT has a moderate correlation [31] with patient-reported tremor but exhibits few correlations with other clinical outcomes, which could relate to the fact that tremor-dominant patients often have a more favorable disease course [35].

In addition to the cross-sectional data described above, the study also includes longitudinal data on mortality for all included patients over an eight-year period, which is considerable in the course of PD. Interpretation of the mortality data is somewhat limited, however, by the lack of supplementary information such as comorbidities, which would permit reliable regression analyses. Judging by SMR, mortality was relatively high compared to other studies [36], including a recent Swedish study of patients with idiopathic PD [37]. This could relate to the fact that study participants were observed in later stages of their disease, since studies with longer observation times usually report higher SMR in patients with PD [36, 38].

Because uncontrolled dyskinesia (DKS > 9) was infrequent, it was not meaningful to compare patients with controlled and uncontrolled dyskinesia. Elevated DKS at the levels commonly seen here seem however to reflect a positive treatment response rather than problematic motor fluctuations.

Interestingly, patients with uncontrolled bradykinesia had higher mortality rates during the eight-year observation period. After direct adjustment for age and sex, the mortality rates were almost doubled among patients with BKS > 25 compared to patients with BKS ≤ 25. Thus, a single measurement of sensor-assessed bradykinesia appears to have a predictive value regarding long-term mortality. One possible explanation for this finding is that a high BKS reflects more severe disease, which in turn leads to higher mortality. Bradykinesia is central to PD pathophysiology [39], and high degrees of clinically assessed bradykinesia are associated with higher mortality [40]. Another possible explanation is confounding by comorbidities. PKG outcomes are based on accelerometer measurements, which can only partially distinguish between bradykinesia related to PD and slowness of movement related to pain, injuries, or other deficits. The group with BKS > 25 could therefore include patients with comorbidities that increase their mortality risk, independently of PD. Lack of physical activity post-diagnosis is a risk factor for mortality among patients with PD [41]. Finally, there is the possibility that the group with BKS > 25 had higher mortality because they were undertreated, considering their lower median LEDD. It is unclear whether dopaminergic treatment reduces mortality in patients with PD, partly because its well-established role in alleviating motor symptoms complicates long-term clinical trials comparing treatment intensities [42], but since it appears to prevent some complications of PD such as falls [43], it is reasonable to assume that an optimized therapy could have at least some effect on mortality.

Assuming that undertreatment influences mortality in PD, our findings would suggest that sensor-assessed bradykinesia could function as a modifiable risk factor for mortality, in analogy with blood pressure in cardiovascular disease [44]. A previous study of 103 patients with PD, where treatment was guided by PKG-derived BKS < 25 as the primary target, indicated that almost 50% patients could be treated towards lower BKS and better clinical outcomes [33].

This study has limitations. Although we aimed to identify a representative sample, we could not reach patients with infrequent clinical visits (less than annually), those managed by private neurologists (uncommon in Sweden), or those managed only by primary care physicians (typically institution-dwelling patients in Sweden). Additionally, the patients who opted not to participate were older than those included in the study, and could have been phenotypically different, e.g., more severely affected in terms of motor impairment or cognitive decline. The latter aspect, however, would rather suggest that our finding of unexpectedly hypokinetic objective measures may be an underestimation. Another limitation is that our clinical evaluations were based on CISI-PD rather than MDS-UPDRS, which is more extensive and could have provided more detailed clinical ratings. Finally, our analysis of mortality data was limited by the lack of supplementary information, such as comorbidity and cause of death, and the lack of longitudinal treatment data.

## Conclusions

This study improves our understanding of wearable sensor measurements by describing the movement patterns of population-representative PD patients in their daily lives, showing that sensor-based motor assessments reflect clinical outcome measures. A high degree of sensor-assessed bradykinesia (BKS > 25) at baseline was associated with higher mortality, shorter disease duration and less intensive treatment. The results suggest that BKS > 25 is a risk factor for mortality, possibly as a marker of undertreatment. Further research is warranted regarding the prognostic implications of sensor-based motor assessments.

## Supplementary Information

Supplementary Material 1.

## Data Availability

The datasets used and/or analyzed during the current study are available from. the corresponding author on reasonable request. With approval from the Swedish Ethical Review Authority and permission from the scientific committee of the Swedish Parkinson Registry, clinical register data for the study participants may also be accessed from the Swedish Parkinson Registry.

## Abbrevations

BKS: Bradykinesia score
CI: Confidence interval
CISI-PD: Clinical Impression of Severity Index for Parkinson’s Disease
COMT: Catechol-O-methyltransferase
DKS: Dyskinesia score
EQ-5D-5L: EuroQoL Five-Dimension Five-Level Scale
FDS: Fluctuation dyskinesia score
HRQOL: Health-related quality of life
LEDD: Levodopa-equivalent daily dose
MAO-B: Monoamine oxidase B
MDS-UPDRS: Movement Disorder Society-Sponsored Revision of the Unified Parkinson’s Disease Rating Scale
NMSQ: Non-Motor Symptoms Questionnaire
PD: Parkinson’s disease
PDQ8-SI: Eight-Item Parkinson’s Disease Questionnaire Summary Index
PKG: Personal KinetiGraph / Parkinson’s KinetiGraph
PRO-PD: Patient-Reported Outcomes in Parkinson’s Disease
PTI: Percent time immobilized
PTT: Percent time with tremor
Q1: First quartile
Q3: Third quartile
r_S_: Spearman’s rank correlation coefficient
SMR: Standardized mortality ratio
