# Supplementary material for: Wearable sensor measurements in relation to clinical characteristics and mortality in patients with Parkinson’s disease
Source: Neurol Res Pract. 2026 Mar 11;8(1):14. doi: 10.1186/s42466-026-00474-8 (PMC12980861; doi:10.1186/s42466-026-00474-8)
Supplement: Supplementary file 1 — Supplementary Material 1. [file 42466_2026_474_MOESM1_ESM.docx]

| **Supplementary Table 1**  PKG measures and continuous clinical variables | | | | | | |
| --- | --- | --- | --- | --- | --- | --- |
|  | Median (Q1–Q3) | Spearman rank correlations with *P* < 0.01* | | | | |
|  |  | BKS | DKS | FDS | PTI | PTT |
| Age, years | 73 (66–78) | 0.24 | -0.19 | -0.25 | 0.22 | - |
| Time since diagnosis, years | 5.3 (2.6–10.4) | - | - | 0.20 | - | -0.16 |
| LEDD, mg | 600 (400–959) | -0.16 | 0.23 | 0.20 | -0.23 | -0.26 |
| CISI-PD, total | 9 (6–11) | 0.21 | - | - | 0.22 | - |
| CISI-PD, disability | 2 (2–3) | 0.34 | -0.25 | -0.16 | 0.30 | - |
| CISI-PD, cognitive status | 1 (0–2) | 0.25 | -0.20 | -0.17 | 0.24 | - |
| PRO-PD, total | 995 (627–1399) | 0.20 | -0.19 | - | 0.21 | - |
| PRO-PD, slowness | 50 (23–65) | 0.25 | -0.23 | - | 0.24 | - |
| PRO-PD, walking | 33 (14–60) | 0.17 | -0.19 | - | 0.17 | - |
| PRO-PD, falling | 5 (0–31) | - | -0.16 | - |  | - |
| PRO-PD, rising from seated position | 24 (7–50) | 0.20 | -0.20 | - | 0.22 | - |
| PRO-PD, dressing/earing/grooming | 17 (4–49) | 0.31 | -0.30 | - | 0.32 | - |
| PRO-PD, motivation/initiative | 24 (5–50) | 0.31 | -0.33 | -0.19 | 0.37 | - |
| PRO-PD, depression | 17 (4–37) | 0.21 | -0.21 | -0.17 | 0.21 | - |
| PRO-PD, loss of interest | 19 (3–48) | 0.26 | -0.27 | - | 0.31 | - |
| PRO-PD, daytime sleepiness | 30 (12–51) | 0.22 | -0.23 | -0.16 | 0.25 | - |
| PRO-PD, dyskinesia | 6 (1–45) | -0.27 | 0.26 | 0.34 | -0.16 | -0.25 |
| PRO-PD, tremor | 46 (8–65) | - | - | - | - | 0.52 |
| PRO-PD, balance | 35 (14–50) | - | -0.18 | - | 0.16 | - |
| PRO-PD, drooling | 12 (2–50) | 0.20 | -0.18 | -0.20 | 0.16 | - |
| PRO-PD, control of body temperature | 48 (19–54) | 0.18 | - | - | - | - |
| PRO-PD, urinary symptoms | 50 (8–77) | - | -0.18 | - | - | - |
| PRO-PD, hallucinations/delusions | 2 (0–18) | 0.17 | -0.16 | - | 0.18 | - |
| NMSQ, total | 10 (6–14) | - | - | - | - | - |
| EQ5D5L, summary index | 0.71 (0.53–0.84) | -0.27 | 0.23 | - | -0.23 | - |
| EQ5D5L, mobility | 1 (0–2) | 0.19 | -0.21 | - | 0.22 | - |
| EQ5D5L, self-care | 1 (0–2) | 0.38 | -0.34 | -0.22 | 0.33 | - |
| EQ5D5L, usual activities | 1 (0–2) | 0.31 | -0.26 | - | 0.30 | - |
| EQ5D5L, anxiety/depression | 1 (0–1) | 0.18 | -0.17 | - | - | - |
| PDQ8, summary index | 25 (13–41) | 0.24 | -0.20 | - | 0.21 | - |
| PDQ8, mobility | 1 (0–2) | 0.19 | -0.20 | - | 0.18 | - |
| PDQ8, activities of daily living | 1 (0–3) | 0.39 | -0.34 | -0.23 | 0.32 | - |
| PDQ8, emotional well-being | 1 (0.3–2) | - | - | - | 0.17 | - |
| PDQ8, social support | 0 (0–1) | - | - | - | - | -0.17 |
| PDQ8, communication | 1 (0–2) | 0.21 | -0.20 | - | 0.17 | - |
| BKS, bradykinesia score; CISI-PD, Clinical Impression of Severity Index for Parkinson’s Disease; DKS, dyskinesia score; EQ-5D-5L, EuroQoL Five-Dimension Five-Level Scale; FDS, fluctuation dyskinesia score; LEDD, levodopa-equivalent daily dose; NMSQ, Non-Motor Symptoms Questionnaire; PDQ8, Eight-Item Parkinson’s Disease Questionnaire; PRO-PD, Patient-Reported Outcomes in Parkinson’s Disease; PTI, percent time immobilized; PTT, percent time with tremor; Q1, first quartile; Q3, third quartile. *Only correlations significant at the 0.01 level (two-tailed) are shown. No correction has been made for multiple comparisons. Scale subitems where no correlation was significant have been omitted. | | | | | | |

|  |  |  |
| --- | --- | --- |

| **Supplementary Table 2**  PKG measures and dichotomous clinical variables | | | | | | |
| --- | --- | --- | --- | --- | --- | --- |
|  | Number, yes/no | Median values with a significant difference, yes/no* | | | | |
|  |  | BKS | DKS | FDS | PTI (%) | PTT (%) |
| Male sex | 184/97 | 31.6/26.2 | 1.0/1.8 | 7.3/8.4 | 9.4/6.1 | - |
| Living alone | 60/221 | - | - | - | - | - |
| Treatment at non-university hospital | 189/92 | - | - | - | - | - |
| Orthostatic hypotension | 36/199 | - | - | - | - | - |
| Advanced treatment** | 15/266 | - | 3.3/1.2 | 9.8/7.5 | 3.8/8.8 | - |
| NMSQ item 1, dribbling | 98/182 | 30.5/28.4 | - | - | 11.1/6.8 | - |
| NMSQ item 7, incomplete bowel emptying | 99/181 | - | - | 8.0/7.2 | - | - |
| NMSQ item 11, weight change | 38/241 | - | 1.8/1.1 | - | - | - |
| NMSQ item 13, loss of interest | 78/201 | - | - | - | 10.6/7.1 | - |
| NMSQ item 19, sex difficulties | 122/149 | - | - | - | 10.5/6.3 | - |
| NMSQ item 21, falling | 86/193 | - | 0.7/1.4 | - | - | - |
| NMSQ item 27, leg swelling | 96/184 | - | - | 7.2/7.9 | - | - |
| NMSQ item 28, excessive sweating | 63/217 | 27.1/30.3 | 1.6/1.1 | - | - | - |
| NMSQ item 29, double vision | 56/224 | 31.1/28.8 | - | - | - | - |
| NMSQ item 30, delusions | 27/253 | - | 0.6/1.2 | - | 14.7/7.8 | - |
| BKS, bradykinesia score; DKS, dyskinesia score; FDS, fluctuation dyskinesia score; NMSQ, Non-Motor Symptoms Questionnaire; PTI, percent time immobilized; PTT, percent time with tremor. *Median values for each group are only presented when the difference is significant at the 0.01 level (Mann-Whitney). No correction has been made for multiple comparisons. NMSQ subitems without significant differences have been omitted. **Deep brain stimulation, levodopa-carbidopa intestinal gel, or subcutaneous apomorphine infusion. | | | | | | |
